# Prevalence of Hepatitis E Virus and Its Associated Outcomes among Pregnant Women in China

**DOI:** 10.3390/pathogens12091072

**Published:** 2023-08-22

**Authors:** Gui-Ping Wen, Min-Ming Wang, Zi-Min Tang, Chang Liu, Zi-Hao Yu, Zheng Wang, Zi-Zheng Zheng, Yu-Lin Zhou, Yun-Sheng Ge

**Affiliations:** 1 Department of Central Laboratory, Women and Children’s Hospital, School of Medicine, Xiamen University, Xiamen 361102, China; wenguiping@xmu.edu.cn (G.-P.W.); zhou_yulin@126.com (Y.-L.Z.); 2 United Diagnostic and Research Center for Clinical Genetics, Women and Children’s Hospital, School of Medicine and School of Public Health, Xiamen University, Xiamen 361102, China; wmmya966@stu.xmu.edu.cn (M.-M.W.); wangz404@stu.xmu.edu.cn (Z.W.); 3 State Key Laboratory of Molecular Vaccinology and Molecular Diagnostics, National Institute of Diagnostics and Vaccine Development in Infectious Diseases, School of Public Health, Xiamen University, Xiamen 361102, China; zimintang@xmu.edu.cn (Z.-M.T.); changl@stu.xmu.edu.cn (C.L.); 17725352695@163.com (Z.-H.Y.); 4 NMPA Key Laboratory for Research and Evaluation of Infectious Disease Diagnostic Technology, School of Public Health, Xiamen University, Xiamen 361102, China; 5 School of Pharmacy, Xiamen University, Xiamen 361102, China

**Keywords:** hepatitis E virus, pregnant women, HEV antigen, outcome, prevalence

## Abstract

Hepatitis E virus (HEV) is a significant public health concern worldwide. Pregnant women are at high risk of severe HEV infection. Various adverse outcomes in pregnant women related to HEV infection have been well documented in low-income and middle-income countries with poor sanitation. However, previous studies have provided inconsistent conclusions regarding the effects of HEV infection on the health of pregnant women and their infants in developed countries and contemporary China. In China, previous studies on HEV in pregnant women mainly focused on anti-HEV IgM and/or anti-HEV IgG. In this study, 4244 pregnant women were retrospectively analyzed for HEV-related markers. The positive rates of HEV antigen, HEV RNA, anti-HEV IgM, and anti-HEV IgG were 0.28%, 0.54%, 0.35%, and 10.49%, respectively. Among the 467 pregnant women who tested positive for at least one HEV-related marker, 92.93% (434) were positive for anti-HEV IgG only and 0.21% (1) were positive for HEV antigen, anti-HEV IgM, and anti-HEV IgG. Although the prevalence of anti-HEV IgG significantly increased with age, the prevalence of anti-HEV IgM, HEV RNA, and HEV antigen did not differ among pregnant women of different ages. Thirty-three pregnant women were positive for at least one of anti-HEV IgM, HEV antigen, and HEV RNA, and these individuals were recently or currently infected with HEV. None of the 33 pregnant women exhibited obvious clinical symptoms. Of the 33 pregnant women, 39.39% (13) experienced adverse fetal outcomes, including preterm birth, fetal distress, and low birth weight, the incidence of which was significantly higher than in pregnant women who were not recently or currently infected with HEV. These findings suggest that maternal HEV infection may impact the health of fetuses; thus, these results may contribute to the development of appropriate public health interventions for this population.

## 1. Introduction

Hepatitis E virus (HEV) is the leading cause of acute viral hepatitis globally. HEV infection is now recognized as a significant public health concern in many developing and developed countries. Approximately 2 billion people reside in areas where hepatitis E is endemic [1]. According to the World Health Organization, there are approximately 20 million HEV infections each year, resulting in 3.3 million symptomatic cases [2]. In 2015, HEV infections accounted for approximately 44,000 deaths, which was 3.3% of deaths due to viral hepatitis. Eight genotypes of HEV have been identified, of which genotypes 1–4 are the major human-pathogenic genotypes. Genotypes 1 (HEV-1) and 2 (HEV-2) exclusively infect humans and are typically transmitted through contaminated drinking water in developing countries, leading to large outbreaks with high morbidity and mortality rates. In contrast, genotypes 3 (HEV-3) and 4 (HEV-4) are zoonotic, infecting humans as well as other mammalian species, with swine serving as the main reservoir. Infections with HEV-3 and HEV-4 are commonly associated with the consumption of raw or undercooked meat (e.g., pork and game) or shellfish, resulting in sporadic cases worldwide. As a result of rapid industrialization and economic development, large outbreaks of HEV have rarely been seen in China after 2000. Currently, HEV-4 is the dominant genotype in China, and the epidemiology of HEV has shifted from an outbreak to a sporadic form [3,4].

In the general population, HEV usually causes acute, self-limiting disease with a mortality rate of 0.2–1% [5]. However, it is crucial to note that certain populations, such as pregnant women, are at particularly high risk of severe HEV infection [6]. The incidence of HEV infection is high during pregnancy, and HEV-infected pregnant women may experience fulminant hepatitis, obstetrical complications, and even death, with mortality rates as high as 30% [6,7]. HEV-infected pregnant women have a higher incidence of fulminant hepatic failure (FHF) than pregnant women with other viral hepatitis [8,9]. The mortality rate in HEV-infected pregnant women was 41%, compared to a considerably lower rate of 7% in pregnant women not infected with HEV [8]. During the HEV outbreaks in China from 1986 to 1988 and Niger in 2017, 57.1% and 44.7% of recorded deaths, respectively, were in pregnant women [4,10]. Surveillance data in Bangladesh suggested that approximately 10% of pregnancy-related deaths were likely attributable to HEV [11]. Additionally, HEV infection in pregnant women can lead to adverse fetal outcomes, including preterm delivery, low birth weight, spontaneous abortions, and stillbirths [8,12]. These adverse outcomes of HEV infections in pregnant women and their fetuses or neonates have been well documented in relation to HEV-1 and HEV-2 in low-income and middle-income countries with poor sanitation, including Bangladesh [13], India [8,12,14], Chad [15], Niger [10], and Sudan [16].

Some studies have explored the impact of sporadic HEV infection on the health of pregnant women and their fetuses or neonates in developed countries and contemporary China, where HEV-3 and HEV-4 infections play a dominating role and lead to sporadic cases [4,17,18,19,20,21,22,23]. However, these studies have provided conflicting conclusions [4,17,18,19,20,21,22]. For instance, Bouthry et al. [20] reported that HEV-infected pregnant women in France exhibited nonspecific symptoms or asymptomatic alanine aminotransferase elevation, and no adverse outcomes were noted in these women or their neonates. Similar findings were observed in previous studies conducted in China [22] and Germany [17]. Nevertheless, recent studies have demonstrated that HEV infections can lead to various adverse outcomes in pregnant women and their fetuses or neonates in China and the United States [4,18,19,21]. Wasuwanich et al. [18] documented that although no maternal or fetus/neonate deaths were reported in the United States, considerable adverse events were observed in HEV-infected pregnant women and their fetuses/neonates. About 84% of HEV-infected pregnant women experienced complications such as polyhydramnios, hemorrhage, and coagulation defects. Additionally, more than 60% of fetuses/neonates experienced complications, including preterm delivery, abnormal heart rate or rhythm, and growth retardation [18]. HEV-seropositive pregnant women had a higher risk of adverse maternal and fetal outcomes than HEV-seronegative pregnant women [19]. Therefore, more research is needed to determine whether sporadic HEV infection during pregnancy leads to adverse outcomes in these regions.

China is an epidemic region for HEV, and the prevalence of HEV among pregnant women deserves the utmost attention. Some studies have reported the prevalence of HEV among pregnant women in China, with the prevalence of anti-HEV IgM and anti-HEV IgG ranging from 0.2–4.11% and 11.1–20.27%, respectively [19,21,22,24]. The available HEV isolates from pregnant women belonged to HEV-4 [19,21]. The prevalence of HEV among pregnant women in China has not been comprehensively explored as previous studies were primarily based on anti-HEV antibody markers (anti-HEV IgM and/or anti-HEV IgG) [19,21,22,24]. In these studies, only pregnant women with positive anti-HEV antibodies were subjected to HEV RNA detection [19,21,22] or all pregnant women were not tested for HEV RNA [24]. In a previous study conducted in the Inner Mongolia Autonomous Region of China, all pregnant women were tested for HEV RNA using a pool of 30 samples and anti-HEV IgM-positive pregnant women were individually tested for HEV RNA [25]. However, adverse outcomes were analyzed only in the anti-HEV IgM-positive and/or anti-HEV IgG-positive pregnant women [25]. Recent studies have demonstrated that the main target of HEV antigen detection is the freely secreted form of ORF2 protein, which is the predominant form of ORF2 protein present in the serum [26,27]. Notably, a similar nonvirion-associated viral antigen, hepatitis B surface antigen (HBsAg), plays a crucial role in the diagnosis of hepatitis B virus infection [28]. Further research is needed to better understand the prevalence of HEV in pregnant women in China and to develop appropriate public health interventions.

In this study, 4244 pregnant women were enrolled and tested for HEV antigen, HEV RNA, anti-HEV IgM, and anti-HEV IgG to investigate HEV infection in pregnant women in China. Additionally, this study aimed to analyze the impact of HEV infection on the health of pregnant women and their fetuses or neonates.

## 2. Materials and Methods

### 2.1. Participants and Samples

Pregnant women were enrolled at the Women and Children’s Hospital, School of Medicine, Xiamen University, Xiamen, China. From April 2019 to December 2019, serum samples were collected from pregnant women in routine prenatal tests in their second trimester. A total of 4244 pregnant women were enrolled in this study and retrospectively tested for HEV antigen and anti-HEV antibodies (anti-HEV IgM and anti-HEV IgG). All serum samples were stored at −20 °C before detection. This study was conducted in accordance with the Declaration of Helsinki and was approved by the Ethics Committee of the Women and Children’s Hospital, School of Medicine, Xiamen University (No. KY-2020-112).

### 2.2. Detection of HEV Antigen

HEV antigen in the serum samples was detected using commercially available enzyme-linked immunosorbent assay (ELISA) kits (Wantai, Beijing, China), which were used for research purposes only. The assay was performed in accordance with the manufacturer’s instructions. In brief, a mixture of 20 μL of diluent and 50 μL of the serum sample were added to the wells of 96-well microplates, followed by an incubation period of one hour at 37 °C. Thereafter, without any washing, 100 μL of HRP-conjugated mAb no. 4 was added directly to the wells and incubated at 37 °C for 30 min to detect the bound antigens. Subsequently, the plates were washed five times with wash buffer and each well was incubated with 100 μL of tetramethylbenzidine (TMB) substrate solution at 37 °C for 15 min. The reaction was terminated with stop solution, and the optical density (OD) value was determined at 450 nm using a microplate reader (Autobio, Zhengzhou, China) with a reference wavelength set at 620 nm. Readings were completed within 10 min after the termination of the reaction.

### 2.3. Detection of Anti-HEV IgM and Anti-HEV IgG

Serum samples were analyzed for anti-HEV IgM and anti-HEV IgG using commercial anti-HEV IgM and anti-HEV IgG ELISA kits (Wantai, Beijing, China), respectively. These anti-HEV IgM and anti-HEV IgG ELISA kits have been employed in various studies [4,19,24,29]. A μ-chain capture immunoassay was used to detect anti-HEV IgM, while the anti-HEV IgG antibody kits were indirect immunoassays with a recombinant HEV PE2 protein containing 213 amino acids as the coating antigen. The assays were performed following the manufacturer’s instructions. Briefly, 10 μL of serum and 100 μL of diluent were introduced into the wells and then incubated at 37 °C for 30 min. Following five rinses with wash buffer, 100 μL of HRP-conjugated HEV antigen solution (for anti-HEV IgM detection) or HRP-conjugated mouse anti-human IgG antibody (for anti-HEV IgG detection) was introduced into the wells and incubated at 37 °C for 30 min. After washing five times with wash buffer, 100 μL of TMB substrate solution was added to each well and incubated at 37 °C for a duration of 15 min. Subsequently, the reaction was halted and the OD was determined. The results for anti-HEV IgM and anti-HEV IgG were recorded as signal-to-cut-off (S/CO) ratios and values of S/CO ≥ 1.0 were considered as positive, in accordance with the manufacturer’s instructions.

### 2.4. Detection of HEV RNA

Serum samples that tested positive for HEV antigen and/or anti-HEV IgM were individually tested for HEV RNA. The remaining samples were tested for HEV RNA using a pool of 12 samples. Positive pools were resolved to individual samples that were then subjected to HEV RNA detection. HEV RNA detection was performed using a real-time reverse transcription PCR (RT-PCR) assay, as previously reported [6,29]. HEV RNA was extracted from 100 μL aliquots of each serum sample using the commercially available GenMag Viral DNA/RNA Isolation Kit (Genmag, Beijing, China). The RNA extraction was conducted with a DOF-964B purification system (Genmag, Beijing, China), in accordance with the manufacturer’s instructions. Five microliters of the extracted nucleic acid were designated for quantification by a commercial one-step RT-PCR kit (Genmagbio, Beijing, China). The quantification was conducted in a 25 μL reaction mixture under the following conditions: 12.5 μL of 2× one-step RT-PCR buffer, 0.2 μL of GenMagScript RT Enzyme Mix, and 200 nM primers and probe. The forward primer, reverse primer, and probe were JVHEVF: 5′-GGTGGTTTCTGGGGTGAC-3′, JVHEVR: 5′-AGGGGTTGGTTGGATGAA-3′, and JVHEVP: 5′-TGATTCTCAGCCCTTCGC-3′, respectively. Reverse transcription was conducted at 50 °C for a duration of 30 min, followed by denaturation at 95 °C for 10 min. Thereafter, the DNA was amplified with 40 cycles, each comprising amplification at 95 °C for 15 s, annealing at 55 °C for 45 s, and extension at 72 °C for 15 s. These real-time RT-PCR assays were performed utilizing the CFX96TM Real-Time System and C1000TM thermal cycler device (Bio-Rad Inc., Hercules, CA, USA). Upon completion of the reactions, real-time RT-PCR data were collected and threshold cycle (Ct) values were calculated by the CFX manager software. To generate standard quantitation curves, the Ct values were plotted against the input copy numbers. The HEV genome copy number present in the serum samples was then determined by comparing with the standard curve of plasmids with known viral copy numbers. The detection limit of real-time RT-PCR was 800 copies/mL.

### 2.5. Statistical Analysis

Data analysis was performed using SPSS statistics (SPSS, Chicago, IL, USA) and GraphPad Prism (GraphPad Software, San Diego, CA, USA). Categorical data are reported as numbers and percentages. Continuous data are presented as the mean ± standard deviation (SD). The chi-square test was used for comparisons of HEV prevalence among pregnant women of different ages and the adverse outcomes between pregnant women with recent or current HEV infection and pregnant women without recent or current HEV infection. The concordances among HEV antigen, HEV RNA, and anti-HEV IgM were calculated using the Cohen’s Kappa test. The *p*-values were calculated by a two-tailed test, and *p*-values of <0.05 were considered statistically significant.

## 3. Results

### 3.1. Prevalence of HEV Antigen and Anti-HEV Antibodies among Pregnant Women

A total of 4244 pregnant women were enrolled in this study. The mean maternal age was 28.18 ± 3.08 years. All pregnant women were in their second trimester of pregnancy, with a mean gestational age of 16.47 ± 1.15 weeks. These pregnant women were retrospectively analyzed for HEV-related markers, including HEV antigen, HEV RNA, anti-HEV IgM, and anti-HEV IgG. Of the pregnant women, 12 (0.28%) were positive for HEV antigen, 15 (0.35%) were positive for anti-HEV IgM, and 445 (10.49%) were positive for anti-HEV IgG (Table 1). Twenty-three (0.54%) pregnant women tested positive for HEV RNA, and the HEV RNA levels ranged from 3.33 × 10^3^ to 5.79 × 10^4^ copies/mL.

We further analyzed the prevalence of HEV antigen, anti-HEV IgM, HEV RNA, and anti-HEV IgG in pregnant women of different ages (Table 1). The positive rates of HEV antigen were 0.00% (0/18), 0.33% (9/2765), and 0.21% (3/1461) in pregnant women aged ≤19, 20–29, and ≥30 years, respectively. The positive rate of HEV antigen did not significantly differ among pregnant women of different ages (*p* = 0.763). The positive rates of HEV RNA were 0.00% (0/18), 0.69% (19/2765), and 0.27% (4/1461) in pregnant women aged ≤19, 20–29, and ≥30 years, respectively. The positive rate of HEV RNA in pregnant women was not affected by age (*p* = 0.209). Although the positive rate of anti-HEV IgM was higher in pregnant women aged ≥30 years (0.55%, 8/1461), the difference was not significant (*p* = 0.299). The prevalence of anti-HEV IgG significantly increased with age in pregnant women (*p* = 0.001) from 5.56% (1/18) for ≤19 years to 12.94% (189/1461) for ≥30 years. Of note, the group of pregnant women aged ≤19 years included only 18 women, which was lower than the numbers of pregnant women aged 20–29 years and ≥30 years.

### 3.2. The Relationship between HEV Serologic Markers and RNA among Pregnant Women

**Figure 1:**
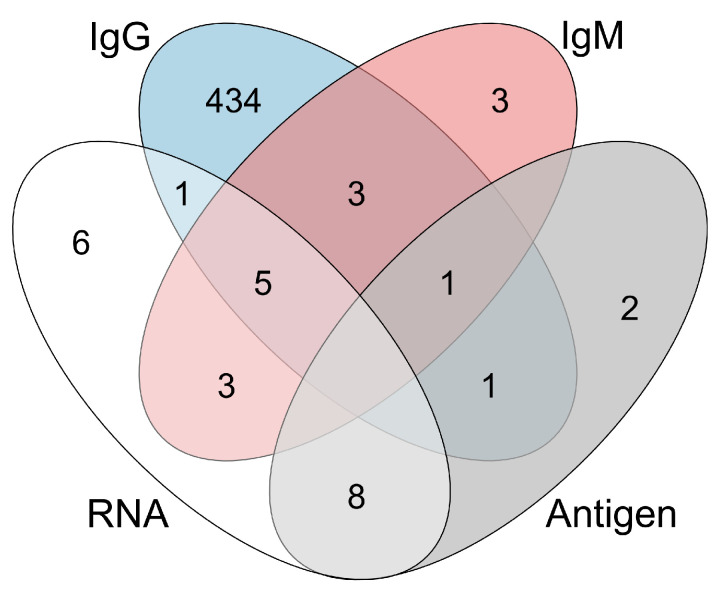
HEV-related marker positivity among pregnant women. IgM: anti-HEV IgM; IgG: anti-HEV IgG; Antigen: HEV antigen; RNA: HEV RNA. The numbers of samples positive for HEV-related markers are indicated in the figure.

In total, 467 samples were positive for at least one HEV-related marker (Figure 1). Among these 467 samples, one was positive for anti-HEV IgG, anti-HEV IgM, and HEV antigen but was negative for HEV RNA. Eight samples were positive for both anti-HEV IgG and anti-HEV IgM but negative for HEV antigen. Among these eight samples, five were positive for HEV RNA. One sample was positive for both anti-HEV IgG and HEV antigen, and one was positive for both HEV RNA and anti-HEV IgG. Three samples were positive for only anti-HEV IgM, and three were positive for both anti-HEV IgM and HEV RNA. Eight were positive for both HEV antigen and HEV RNA, and two were positive for HEV antigen alone. Additionally, six samples were positive for HEV RNA but negative for other HEV-related markers. Furthermore, 434 pregnant women were single-positive for anti-HEV IgG, indicating past HEV infection. Among anti-HEV IgM-positive samples, 60.00% (9/15) were positive for anti-HEV IgG, 6.67% (1/15) were positive for HEV antigen, and 53.33% (8/15) were positive for HEV RNA (Table 2). Among HEV antigen-positive samples, 91.67% (11/12) and 83.33% (10/12) tested negative for anti-HEV IgM and anti-HEV IgG, respectively. To further analyze the relationships between anti-HEV IgM, HEV RNA, and HEV antigen, the concordances among these three tests were calculated using Kappa’s coefficient. The concordance between HEV antigen and anti-HEV IgM was slight (Kappa = 0.07) (Table 2). The concordances between HEV antigen and HEV RNA or HEV RNA and anti-HEV IgM were moderate (HEV antigen and HEV RNA: Kappa = 0.46; HEV RNA and anti-HEV IgM: Kappa = 0.42).

### 3.3. The Potential Impact of Maternal HEV Infections on the Health of Pregnant Women and Their Fetal/Neonatal Outcomes

A total of 33 pregnant women were positive for at least one of HEV antigen, HEV RNA, and anti-HEV IgM. These pregnant women were recently or currently infected with HEV. None of the 33 pregnant women presented obvious clinical symptoms, and no cases of liver failure or death were observed. We further analyzed the fetal/neonatal clinical outcomes in these 33 pregnant women (Table 3). Among the fetuses/infants of these 33 pregnant women, 39.39% (13/33) had adverse fetal/neonatal outcomes. The proportion of adverse outcomes in these pregnant women was significantly higher than that in pregnant women without recent or current HEV infection (23.68%, 997/4211) (*p* = 0.035). The most common outcome in pregnant women with recent or current HEV infection was premature rupture of membranes, which was reported in 30.30% (10/33) of pregnant women. The frequency of premature rupture of membranes in these pregnant women was significantly higher than in pregnant women without recent or current HEV infection (*p* = 0.017). Among pregnant women with recent or current HEV infection, preterm birth was observed in two cases, with deliveries occurring at gestational weeks 35 and 35 + 3, respectively. Fetal distress (abnormal fetal heart monitoring) was observed in two cases, and low birth weight was observed in three cases.

## 4. Discussion

In 2016, the 69th World Health Assembly adopted the “Global Health Sector Strategy on Viral Hepatitis 2016–2021”. The strategy aims to eliminate viral hepatitis as a threat to public health by 2030. Although all hepatitis (hepatitis A, B, C, D, and E) viruses can cause harm to the mother and child, HEV or hepatitis A virus (HAV) infection poses the greatest threat to maternal health and, subsequently, to the fetus during pregnancy [30]. Among patients with sporadic acute viral hepatitis, the proportion of pregnant women in the HEV group was about five-fold higher than in the non-HEV group (HEV group vs. non-HEV group: 31.7% vs. 5.3%) [9]. Considering that pregnant women represent a high-risk population for severe disease after HEV infection, it was of great significance to investigate HEV prevalence in this population. In low-income and middle-income countries with poor sanitation, HEV-1 and HEV-2 infections have been well reported to lead to various adverse maternal and infant outcomes. However, previous studies have provided inconsistent conclusions about the influence of HEV infection on maternal and infant outcomes in China and some developed countries, where sporadic cases of indigenous HEV caused by HEV-3 and HEV-4 predominate [4,17,18,19,20,21,22]. Whether HEV infection in these regions can lead to adverse outcomes during pregnancy is still unclear, and more studies are needed to elucidate this important question.

The anti-HEV IgG seroprevalence in this study was 10.49%, which was slightly higher than the values documented in Argentina (8.4%) [31] and France (7.74%) [29] but lower than previous observations in China (11.1%–20.3%) [19,21,22,24], Ghana (12.2%) [32], and Thailand (41.2%) [33]. The anti-HEV IgM-positive rate was 0.35%, higher than in Yunnan, China (0.2%) [19] and Ghana (0.2%) [32] but lower than those in Jiangsu (0.6%) and Shandong (2.6%), China [22,24] and Thailand (11.8%) [33]. These differences may be attributed to the number of study samples, diagnostic methods, sanitation levels, occupation, and regional differences in HEV prevalence. Our study also revealed that the seroprevalence of anti-HEV IgG increased significantly with age, consistent with previous observations in China [19] and Ghana [32]. This may be associated with the cumulative incidence over time. It is worth noting that there were only 18 pregnant women aged ≤19 years in this study, with the majority of pregnant women aged 20–29 years. The anti-HEV IgM-positive rate was not affected by age in the present study, differing from findings in previous studies [19,33]. Previous studies concerning the prevalence of HEV were primarily performed based on anti-HEV antibodies [4,19,21,22,24,29,31,33]. In this study, all pregnant women were also tested for HEV antigen and HEV RNA. We found that the majority of pregnant women who tested positive for HEV antigen and HEV RNA showed negative results for anti-HEV antibodies.

HEV infection during pregnancy causes various adverse maternal and neonatal outcomes in low-income and middle-income countries where sanitation is poor and HEV infection frequently leads to outbreaks [8,10,12,13,14,15,16]. In this study, no severe disease or maternal mortality was observed in HEV-infected pregnant women. This was in contrast to previous reports that high mortality was observed in pregnancy in large hepatitis E outbreaks in low-income and middle-income countries [8,10,12,13,14,15,16] but was similar to previous observations in China [4], France [20], and the United States [18]. Recently, HEV-related fetal or neonatal adverse outcomes have been reported in the United States and China [4,18,19,21]. In the United States, it was documented that a high proportion of adverse events were experienced by the fetuses/neonates of HEV-infected pregnant women [18]. Approximately 25.00% of the births were preterm births [18], which was significantly higher than the frequency of preterm births reported by the National Center for Health Statistics (9.6%) in the United States. In China, the incidence of adverse outcomes in both anti-HEV IgM- and anti-HEV IgG-positive pregnant women was 72.22% [21]. A recent study reported that 42.99% of the births of HEV-infected pregnant women had adverse fetal or neonatal outcomes in Shanghai, China, including preterm births, stillbirths, and fetal distress [4]. In this study, we also observed that adverse fetal outcomes occurred in pregnant women with recent or current HEV infection, consistent with previous findings [4,18,19,21]. The proportion of adverse fetal outcomes in pregnant women with recent or current HEV infection was significantly higher than in pregnant women without recent or current HEV infection. These results suggest that maternal HEV infections seem to threaten the health of fetuses/infants. HEV has been shown to infect human decidua and placenta, inducing inflammation in these tissue explants [34]. The uterus, the most important organ for pregnancy maintenance, was identified as an extrahepatic site for HEV replication [35]. The disruption of the maternal–fetal interface by HEV replication and the presence of HEV replication in the uterus may contribute to adverse fetal/neonatal outcomes [34,35]. In addition, hormonal changes (including progesterone and estrogen) and suppression of the immune system (e.g., diminished cellular immunity) may also play an important role in adverse fetal/neonatal outcomes during pregnancy [19,36].

Considering that there are no recommended medications for HEV infection in pregnant women, preventive measures against HEV infection in this population may be of great importance. The hepatitis E vaccine (HEV 239) was proven to be safe, effective, and highly immunogenic [37] and has been approved in China and Pakistan. In a phase III clinical trial with more than 100,000 participants, the vaccine had shown an efficacy of 100% within 12 months following the administration of the third dose and could provide protection against HEV-1 and HEV-4 [37]. Furthermore, the vaccine appears to be safe and well-tolerated in pregnant women as the rates of adverse reactions in pregnant women were similar to those in matched nonpregnant women, with no serious adverse events reported [38]. In a rabbit model, the HEV 239 vaccine was able to prevent adverse outcomes caused by multiple genotypes of HEV during pregnancy [39]. Thus, the HEV vaccine may have great potential to reduce the mortality and other adverse outcomes associated with HEV in pregnant women. Nevertheless, the effectiveness and tolerance of this vaccine in this population needs to be fully evaluated.

In this study, we demonstrated the prevalence of HEV in pregnant women in China and observed that most HEV antigen-positive pregnant women were negative for anti-HEV antibodies but positive for HEV RNA. Our results suggest that maternal HEV infection threatens the health of fetuses/neonates. More attention should be given to HEV infection in pregnant women. These findings contribute to providing appropriate public health recommendations for this population.

## Figures and Tables

**Table 1:** Prevalence of HEV antigen, HEV RNA, anti-HEV IgM, and anti-HEV IgG according to the age of the pregnant woman.

Age (Years)	*n*	HEV RNA		HEV Antigen		Anti-HEV IgM		Anti-HEV IgG	
		*n*	Positive Rate (%)	*n*	Positive Rate (%)	*n*	Positive Rate (%)	*n*	Positive Rate (%)
≤19	18	0	0.00%	0	0.00%	0	0.00%	1	5.56%
20–29	2765	19	0.69%	9	0.33%	7	0.25%	255	9.22%
≥30	1461	4	0.27%	3	0.21%	8	0.55%	189	12.94%
Total	4244	23	0.54%	12	0.28%	15	0.35%	445	10.49%
*p*-value			0.209		0.763		0.299		0.001

**Table 2:** Analysis of the Kappa consistency index among HEV antigen, HEV RNA, and anti-HEV IgM.

		HEV Antigen			Kappa (95% CI)	HEV RNA			Kappa (95% CI)
		Positive	Negative	Total		Positive	Negative	Total	
Anti-HEV IgM	Positive	1	14	15	0.07 (−0.07–0.21)	8	7	15	0.42 (0.22–0.62)
	Negative	11	4218	4229		15	4214	4229	
	Total	12	4232	4244		23	4221	4244	
HEV RNA	Positive	8	15	23	0.46 (0.25–0.66)				
	Negative	4	4217	4221					
	Total	12	4232	4244					

**Table 3:** Fetal/neonatal outcomes in pregnant women with recent or current HEV infection.

Variable	HEV RNA-Positive (*n* = 23)	HEV RNA-Negative (*n* = 10)	Pregnant Women with Recent or Current HEV Infection (*n* = 33)	Pregnant Women without Recent or Current HEV Infection (*n* = 4211)	*p* ^1^
Preterm birth, *n* (%)	2 (8.70)	0 (0.00)	2 (6.06)	148 (3.51)	0.430
Threatened preterm labor, *n* (%)	0 (0.00)	0 (0.00)	0 (0.00)	1 (0.02)	0.929
Fetal distress, *n* (%)	2 (8.70)	0 (0.00)	2 (6.06)	164 (3.89)	0.523
Premature rupture of membranes, *n* (%)	7 (30.43)	3 (30.00)	10 (30.30)	643 (15.27)	0.017
Neonatal asphyxia, *n* (%)	0 (0.00)	0 (0.00)	0 (0.00)	24 (0.57)	0.664
Low birth weight, *n* (%)	2 (8.70)	1 (10.00)	3 (9.09)	133 (3.16)	0.054
Macrosomia, *n* (%)	0 (0.00)	0 (0.00)	0 (0.00)	111 (2.64)	0.345
Threatened abortion, *n* (%)	0 (0.00)	0 (0.00)	0 (0.00)	3 (0.07)	0.878
Spontaneous abortion, *n* (%)	0 (0.00)	0 (0.00)	0 (0.00)	14 (0.33)	0.740
Stillbirths, *n* (%)	0 (0.00)	0 (0.00)	0 (0.00)	5 (0.12)	0.843
Without adverse pregnancy outcome, *n* (%)	13 (56.52)	7 (70.00)	20 (60.61)	3214 (76.32)	0.035

^1^ The differences were calculated between pregnant women with recent or current HEV infection and pregnant women without recent or current HEV infection.

## Data Availability

The data supporting the reported results of this article will be made available by the authors.
